# Mutant TDP-43 within motor neurons drives disease onset but not progression in amyotrophic lateral sclerosis

**DOI:** 10.1007/s00401-017-1698-6

**Published:** 2017-03-29

**Authors:** Dara Ditsworth, Marcus Maldonado, Melissa McAlonis-Downes, Shuying Sun, Amanda Seelman, Kevin Drenner, Eveline Arnold, Shuo-Chien Ling, Donald Pizzo, John Ravits, Don W. Cleveland, Sandrine Da Cruz

**Affiliations:** 1 Ludwig Institute for Cancer Research, University of California, San Diego, 9500 Gilman Drive, La Jolla, CA 92093-0670 USA; 2 Department of Cellular and Molecular Medicine, University of California, San Diego, 9500 Gilman Drive, La Jolla, CA 92093-0670 USA; 3 Department of Pathology, University of California, San Diego, La Jolla, CA 92093 USA; 4 Department of Neurosciences, University of California, San Diego, La Jolla, CA 92093 USA; 5 Department of Pathology, Johns Hopkins University, Baltimore, MD 21205 USA; 6 Department of Physiology, National University of Singapore, Singapore, 117549 Singapore; 7 Program in Neuroscience and Behavior Disorders, Duke-NUS Graduate Medical School, Singapore, Singapore

**Keywords:** Amyotrophic lateral sclerosis (ALS), TDP-43, Frontotemporal dementia (FTD), RanGAP1, Motor neuron, Non-cell autonomous, Neurodegeneration, Mouse model

## Abstract

Mutations in TDP-43 cause amyotrophic lateral sclerosis (ALS), a fatal paralytic disease characterized by degeneration and premature death of motor neurons. The contribution of mutant TDP-43-mediated damage within motor neurons was evaluated using mice expressing a conditional allele of an ALS-causing TDP-43 mutant (Q331K) whose broad expression throughout the central nervous system mimics endogenous TDP-43. TDP-43^Q331K^ mice develop age- and mutant-dependent motor deficits from degeneration and death of motor neurons. Cre-recombinase-mediated excision of the TDP-43^Q331K^ gene from motor neurons is shown to delay onset of motor symptoms and appearance of TDP-43-mediated aberrant nuclear morphology, and abrogate subsequent death of motor neurons. However, reduction of mutant TDP-43 selectively in motor neurons did not prevent age-dependent degeneration of axons and neuromuscular junction loss, nor did it attenuate astrogliosis or microgliosis. Thus, disease mechanism is non-cell autonomous with mutant TDP-43 expressed in motor neurons determining disease onset but progression defined by mutant acting within other cell types.

**Electronic supplementary material:**

The online version of this article (doi:10.1007/s00401-017-1698-6) contains supplementary material, which is available to authorized users.

## Introduction

Amyotrophic lateral sclerosis (ALS) and frontotemporal dementia (FTD) are progressive adult-onset neurodegenerative diseases with overlapping clinical and pathological features [34]. In both human patients and animal models of disease, motor neurons appear to be selectively vulnerable to age-dependent degeneration, despite nearly ubiquitous expression of an ALS-associated mutation in multiple cell types. Many hypotheses have been proposed to explain the selective vulnerability of motor neurons [34, 63], including a reduced level of relevant chaperone proteins that allows the accumulation of misfolded protein in motor neurons, relative to other cell types [15, 26, 62].

Expression in mice of ALS-linked mutations in ubiquitously expressed superoxide dismutase (SOD1) produces progressive motor neuron disease. Using either Cre-recombinase mediated genetic approaches or delivery of a short hairpin RNA (shRNA) [16] or a microRNA [66] to lower mutant SOD1 synthesis, the age of disease onset is delayed by reduction of mutant SOD1 expression within motor neurons [9, 69] and precursors of oligodendrocytes [30], while rate of disease progression is delayed by reducing mutant SOD1 synthesized by microglia [5, 9] or astrocytes [16, 67, 69]. A direct non-cell autonomous toxicity from astrocytes towards motor neurons has also been shown in multiple in vitro co-culture systems using astrocytes expressing mutant SOD1 or derived from ALS patients [7, 14, 21, 39, 50].

A majority (~90%) of ALS cases occurs sporadically with no known familial pattern of inheritance or associated gene mutation. Importantly, pathological comparisons between sporadic and inherited cases have revealed a common biochemical signature of ubiquitinated lesions containing TAR DNA binding protein (TDP-43) [1, 44], with the appearance of TDP-43-positive inclusions in almost all ALS cases, except in those caused by mutation in SOD1 or FUS [36, 56]. The discovery of ALS-linked mutations in TDP-43 within a subset of familial cases demonstrated a causative role for this protein in disease pathogenesis [12, 60].

Similar to the ubiquitously expressed SOD1, TDP-43 is expressed in most cell types. Whether TDP-43 mis-accumulation leads to pathology affecting the same cell types as those affected by SOD1 mutants is not known. Previous studies have provided conflicting evidence for a potential non-cell autonomous contribution of mutant TDP-43 to motor neuron degeneration. Neuronal inducible overexpression of TDP-43^M337V^ was sufficient to cause rapidly fatal disease in rats [23], demonstrating an apparent cell-intrinsic component to toxicity mediated by high levels of TDP-43. However, the same team reported that restricted overexpression of TDP-43^M337V^ induced in astrocytes was also sufficient to cause motor neuron degeneration in vivo [64] and led to upregulation of neurotoxic factors in vitro [22]. Despite this, astrocytes derived from glial precursor cells isolated from mice expressing TDP-43^A315T^ at threefold higher than endogenous TDP-43 in the central nervous system were not toxic to wild-type motor neurons in an in vitro co-culture assay [20]. Since TDP-43 protein aggregates appear in the majority of sporadic ALS cases, understanding the cell type specific contributions of TDP-43 to disease pathogenesis has broad implications that could direct therapeutic design for more than the subset of familial patients carrying specific mutations.

Our prior work established that ALS-linked mutant TDP-43^Q331K^ causes progressive age-dependent motor neuron degeneration without a requirement for nuclear-cytosolic relocalization or protein aggregation [2]. Rather, moderate level expression of mutant TDP-43^Q331K^ led to widespread splicing defects throughout the spinal cord and brain. Mutant TDP-43^Q331K^ expressing animals developed age-dependent lower motor neuron disease, progressive decline in motor performance, and reduced hindlimb strength, accompanied by ~30% loss in innervated neuromuscular junctions, motor axons, and motor neurons.

Given the motor defects observed in mice expressing mutant TDP-43^Q331K^ broadly throughout the central nervous system, we have now tested whether mutant TDP-43^Q331K^ synthesized within motor neurons drives disease onset or progression. Selective excision of the mutant encoding TDP-43^Q331K^ transgene from motor neurons is found to delay age of disease onset with no effect on disease progression after onset despite complete preservation of motor neuron numbers even at late ages. Glial cell activation was unaffected, consistent with mutant TDP-43 expression within glial cells and/or other surrounding neurons contributing to non-cell autonomous motor neuron degeneration and death.

## Materials and methods

### Generation of transgenic mice expressing mutant TDP-43^Q331K^ throughout the central nervous system, with levels selectively lowered in motor neurons

Transgenic mice which express a deletable human TDP-43 transgene carrying a mutation at Q331K broadly within the central nervous system were described in [2] and deposited at Jackson Laboratories as B6N.Cg-Tg(Prnp-TARDBP*Q331K)103Dwc/J. For selective expression of Cre recombinase in motor neurons, VChAT-Cre mice in a C57Bl6 background [40, 69] were bred to the floxed TDP-43^Q331K^ line 103. To assess the specificity of Cre activity within motor neurons, mice were bred to Rosa-26 reporter mice which ubiquitously express a β-galactosidase transgene that can only be translated upon Cre-mediated recombination to remove a premature stop cassette, also in a C57Bl6 background [59, 69]. Breeding schemes paired TDP-43^Q331K^ animals with double transgenic Rosa-26/VChAT-Cre animals to produce litters with triple transgenic offspring. Upon tissue collection at various ages, genotypes for all animals were reconfirmed using fresh tail biopsies.

### RNA extraction and RT-qPCR

Total RNA was isolated using Trizol (Invitrogen) extraction, and mRNA levels were determined by qRT-PCR using the iQSYBR Green supermix (Bio-Rad).

### Protein extraction and immunoblotting

Tissues were homogenized in a glass dounce homogenizer in lysis buffer (800 μL per spinal cord) (20 mM Hepes pH 7.4, 5 mM MgCl_2_, 150 mM KCl, 0.5 mM DTT, with freshly added protease inhibitors and RNAseOUT). An aliquot of the lysate was set aside for RNA extraction using trizol, while the rest was processed for protein extraction. Lysates were centrifuged at 2000 g for 10 min to remove myelin, the lysis buffer was supplemented with NP-40 for a final concentration of 1% NP-40, and then centrifuged at high speed, 15,000 rpm, for 15 min. Equal amounts of each sample (30 μg protein) were loaded onto a 12.5% Tris–HCl gel (Bio-Rad, Criterion). Following transfer to nitrocellulose, the immunoblot was sequentially probed with an antibody that recognizes both human and mouse TDP-43 (Proteintech #12892, 1:1000), the Myc-tag (Millipore 4A6, 1:1000), or Hsp90 (Cell Signaling C45G5, 1:1000).

### Immunofluorescence

Tissue preparation for immunofluorescence was performed as previously described [2]. Anesthetized animals were transcardially perfused with 4% (vol/vol) paraformaldehyde in phosphate-buffered saline pH 7.4, then tissues were cryopreserved in 30% (wt/vol) sucrose, and embedded in TissueTek OCT (Sakura). Following cryosectioning of spinal cord into 30 μm-thick sections, free floating sections were selected from the same well per animal for each experiment and immunostained with the indicated antibodies. In brief, free floating sections were washed in PBS three times before incubation in blocking solution containing PBS with 1.5% (wt/vol) BSA and 0.5% Tween-20 for 1 h at room temperature. Sections were incubated in primary antibodies (detailed in Supplementary Materials) in PBS, 0.3% (wt/vol) Triton-X 100 overnight at room temperature, and then washed three times in PBS before incubation with secondary antibodies in PBS, 0.3% (wt/vol) Triton-X 100 for 1 hr at room temperature. After incubation with DAPI (1 μg/ml) and a final wash with PBS, sections were mounted onto glass slides and allowed to dry overnight before coverslipping with ProLong Gold anti-fade mounting media (Invitrogen). For detection of primary antibodies, donkey anti-rabbit, anti-mouse, or anti-goat Cy3, Cy5, or Alexa-488 conjugated antibodies (Jackson ImmunoResearch) were used at a 1:500 dilution.

### Immunohistochemistry

Tissue sections were cut from blocks of formalin-fixed paraffin embedded ALS tissue, obtained from Dr. John Ravits’ bank collection (control patient #83 and #65). The spinal cord sections of patients harboring TDP-43 mutations (Pt #284 and #203) as well as control #150 were obtained from the MRC London Neurodegenerative Diseases Brain Bank (King’s College London) (see table below). Seven µm-thick tissue sections were stained with an antibody against RanGAP1 (ab92360) from Abcam (Cambridge, MA, USA). Slides were stained on a Ventana Discovery Ultra (Ventana Medical Systems, Tucson, AZ, USA). Antigen retrieval was performed using Tris–EDTA based cell conditioning solution (CC1) for 40 min at 95 °C. The primary antibody was incubated on the sections at 1:300 dilution for 32 min at 37 °C followed by UltraMap (Ventana) and DAB detection. Slides were rinsed, dehydrated through alcohol and xylene, and coverslipped. Imaging was performed on Nanozoomer slide scanner (Hamamatsu) at the UCSD microscopy core.

**Table:** 

Patient #	Age at death (years)	Sex	Diagnosis	Genotype	Post-mortem delay + interval (h)	Reference
65	82	M	NORMAL	N/A	4*	
83	63	F	NORMAL	N/A	4*	
A150/01	40	M	NORMAL	N/A	40	
A284/13	57	M	ALS	TARDBP M337V	74	[60]
A203/12	23	F	ALS	TARDBP Y374X + FUS P525L	125	[32]

* Post-mortem interval only (post-mortem delay not available)

### Animal behavior

Animal studies were carried out under protocols approved by the Institutional Animal Care and Use Committee of the University of California San Diego (IACUC protocol #S00225), and were in compliance with the Association for Assessment of Laboratory Animal Care guidelines for animal use. For behavior experiments, TDP-43^Q331K^ and TDP-43^Q331K^/VChAT-Cre animals were compared to contemporaneously produced littermates, either containing or lacking the Rosa26-LacZ reporter.

#### Accelerating rotarod

To test motor performance, cohorts of age-matched, gender-matched transgenic animals and littermate controls were tested for the time to fall on an accelerating rotarod apparatus (UGO Basile; 2–40 rpm). Animals were tested over three trials with a maximum time of 300 s per trial at each age (3, 6, 10–12, 15–16, ≥18 months). Latency to fall was recorded in seconds once the mice fell from the bar, or rotated once around the bar, according to previous studies [2, 10, 37]. Following a 1-day training session, motor performance was assessed in three trials each day over the course of three consecutive days. The data shown are the average of nine values per animal across the three sessions, ±SEM from *n* ≥ 4 animals per group at each time point. Animals were studied longitudinally over time, and the graph in Fig. 2a (and Supplementary Fig. 2a) depicts the same animals followed from 3- to 15–18 months of age, with a reduced *n* per group over time as animals were collected for tissue analysis.

#### Analysis of hindlimb clasping

An observer blinded to the genotypes scored animals as being either affected or unaffected by hindlimb clasping. Mice were classified as displaying hindlimb clasping if they displayed retraction of the hindlimbs after being lifted by the tail for 30 s or less. Prior to tissue collection, all animals were video recorded to document hindlimb clasping and other phenotypes. A percentage of animals affected at 10–12 or 19–24 months is shown in Fig. 2b, with *n* ≥ 12 per group.

### Quantification of lower motor neurons

ChAT-positive ventral horn motor neurons were quantified from at least 20 sections per animal, spaced 360 μm apart throughout the lumbar spinal cord. Data were acquired from *n* > 3 animals per genotype at 10–12 or 19–24 months of age and are shown as the average of the total number of motor neurons counted divided by the number of sections for each animal, ±SEM.

### Morphometric analysis and quantification of L5 root motor axons

Mice were perfused transcardially with 4% paraformaldehyde in 0.1 M Sorenson’s phosphate buffer, pH 7.4. Roots from lumbar level 5 of the spinal cord (L5) were dissected from *n* > 3 animals per genotype at 10–12 months and at 19–24 months of age, and preserved in fixative at 4 °C until embedding. Roots were embedded in Epon-Araldite as previously described in [47]. Thick sections (0.75 μm) were prepared and stained with toluidine blue for analysis by light microscopy. For animals in each age group, cross sections of axons were analyzed using BioQuant software and axonal diameters were graphed as a size distribution curve.

### Quantification of neuromuscular junctions and morphology

Floating 40 μm-thick longitudinal sections of gastrocnemius muscle were incubated in blocking solution [PBS, 0.5% vol/vol Tween 20, 1.5% wt/vol BSA] for 4 h at room temperature. Sections were then incubated with polyclonal synaptophysin antibody (ThermoFisher, SP11, 1:50 dilution) in PBS, 0.3% Triton-X 100 at room temperature overnight. The following day, sections were washed in PBS and incubated with donkey anti-rabbit Cy3 (Jackson ImmunoResearch) and α-bungarotoxin Alexa488 (ThermoFisher, B13422) at 1:500 for 1 h, and then with fluoromyelin red (ThermoFisher, F34652) at 1:300 for 30 min. The sections were further washed with PBS, mounted, and dried overnight before coverslipping. For each animal, about ten sections were analyzed for quantification of neuromuscular junctions (shown as average ± SEM). Morphology was scored from >300 NMJs per animal, and endplates that appeared fragmented or small were scored as abnormal. An observer blinded to animal genotype performed quantification and morphology scoring. Representative images were acquired across a 25-μm Z-stack acquired at 20× magnification using a Nikon Eclipse laser scanning confocal microscope, and maximum projection images were obtained using Nikon Elements software (shown in Fig. 3c).

### Confocal microscopy and image analysis

**Fig. 1:**
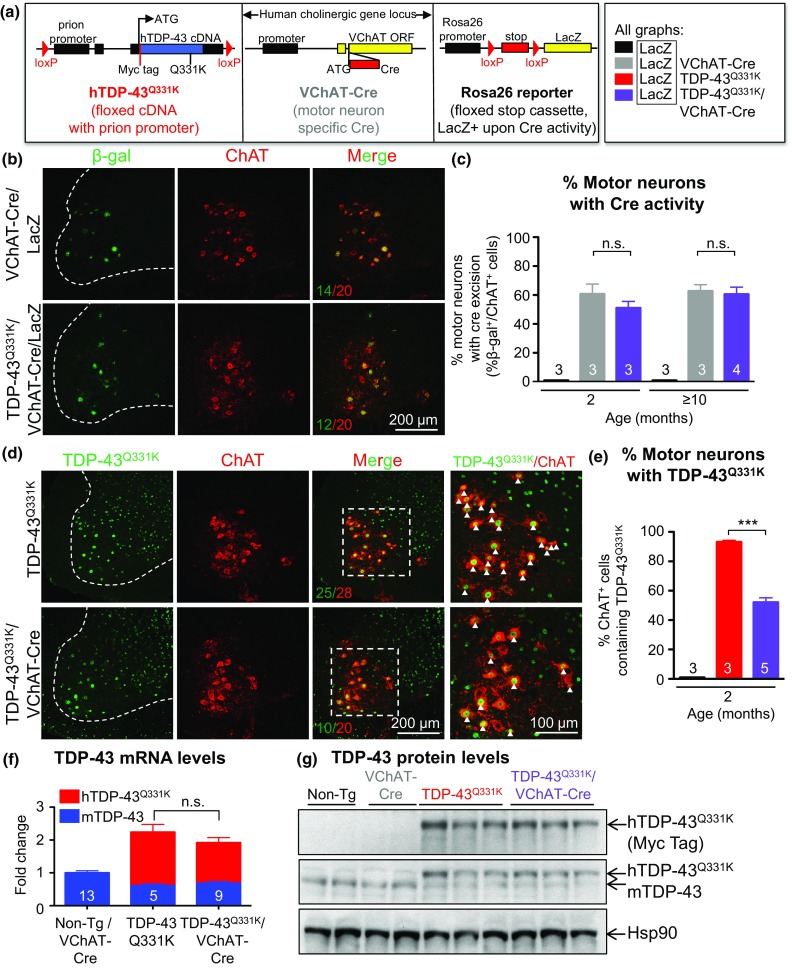
Reduction of mutant TDP-43 in motor neurons without altering total transgene levels. **a** Scheme of the three transgenic mouse lines used in this study: a floxed construct expressing mutant TDP-43^Q331K^ throughout the nervous system driven by the prion promoter, VChAT-Cre targeting Cre to motor neurons, and the Rosa26 reporter to detect Cre activity. **b** Enzymatic Cre activity occurs specifically within lumbar spinal cord motor neurons, as shown by representative micrographs illustrating immunostaining with antibodies against β-galactosidase (β-gal) and ChAT in 2-month-old animals. *Scale bar* 200 µm. **c** Quantification of ChAT positive motor neurons that express β-gal in (**b)**. Cre-excision occurred in about 50% of motor neurons in both VChAT-Cre and TDP-43^Q331K^/VChAT-Cre groups (*n* ≥ 3 animals per group). **d** Representative confocal micrographs of the ventral horn of lumbar spinal cord from 2-month-old animals showing TDP-43^Q331K^ in motor neurons using antibodies against the transgene encoded Myc tag and ChAT. *Scale bar* 200 µm for all images, with enlarged inset panels on the *right*. **e** The percentage of ChAT positive cells containing TDP-43^Q331K^ (*white arrows*) was quantified from *n* ≥ 3 animals per genotype. About 45% of motor neurons show reduced TDP-43^Q331K^ expression in TDP-43^Q331K^/VChAT-Cre animals. **f**, **g** Total levels of TDP-43^Q331K^ are similar between TDP-43^Q331K^ and TDP-43^Q331K^/VChAT-Cre groups at 2 months of age, supporting the selective expression of Cre within motor neurons. **f** mRNA levels of human and mouse TDP-43 were assessed by qRT-PCR using species-specific primers, shown as fold change over controls (Non-Tg and VChAT-Cre) (*n* ≥ 5 per group). **g** Protein levels of human and mouse TDP-43 in total spinal cord lysates were determined by immunoblotting with an antibody recognizing both human and mouse TDP-43

All confocal images were acquired on a Nikon Eclipse laser scanning confocal microscope using Nikon Elements software. For each immunostaining experiment, confocal acquisition parameters were determined optimally below saturation level and kept consistent across all samples. Images used for quantification in Figs. 1c, e, 4b, c, and 5f, g were acquired at 20× magnification. Representative images in Figs. 4a, 5a–e (right panels) were acquired at 60× magnification.

#### VChAT-Cre excision efficiency determined by β-gal/ChAT or Myc/ChAT staining

Co-localization of β-gal or Myc with ChAT staining was determined from multiple 10 μm-thick Z-stack images of the lumbar spinal cord acquired at 20× magnification on a Nikon confocal microscope. The percent of ChAT-positive cells containing β-gal staining were counted from >10 maximum projection images taken across the lumbar region, scoring at least 200 cells per animal in *n* ≥ 3 mice per group. The percent of ChAT positive cells containing bright nuclear Myc (human TDP-43^Q331K^) staining was analyzed using Volocity software to quantify fluorescence intensity of Myc within each ChAT positive cell. An average of 230 ChAT positive cells were counted per animal and scored as either Myc positive or negative relative to a standard Myc fluorescence intensity threshold across samples. To determine percentages of both β-gal/ChAT and Myc/ChAT overlap, *n* ≥ 3 animals were analyzed per group at each time point.

#### Extent of nuclear morphology aberrations based on RanGAP1/Myc/ChAT staining

To assess nuclear morphology in motor neurons, lumbar spinal cord sections were immunostained with RanGAP1, Myc, and ChAT antibodies. Identical observations were made for RanGAP1 aberrations using two different antibodies for RanGAP1 (rabbit polyclonal H-180 and goat polyclonal N-19 from Santa Cruz Biotechnology) and Myc (mouse monoclonal 4A6 from Millipore or rabbit polyclonal from Sigma). For each animal, between 5 and 9 sections of the lumbar spinal cord were imaged using a 20× objective for acquisition across a 10 μm-thick Z-stack. Maximum projection images were scored by an observer blinded to genotypes. All ChAT positive motor neurons were first counted in each image, then scored as containing normal or aberrant nuclear morphology, based on RanGAP1 staining, and scored as positive or negative for Myc levels (transgene) from four to six sections per animal (*n* > 50 cells each, *n* > 4 animals per group). Nuclear morphological aberrations were defined by lack of circularity of the nucleus and/or presence of invaginations or wrinkles around the nuclear perimeter using the RanGAP1 antibody. Representative images for Fig. 4a and Supplementary Fig. 3b were acquired at 60× magnification across a 15 μm-thick Z-stack, and a maximum projection image is shown.

#### Astrogliosis and microgliosis determined by GFAP or Iba1 fluorescence intensity

For quantification of GFAP and Iba1 staining, at least four 20× magnification Z-stack images were analyzed throughout the ventral horn of each animal. Nikon Elements Software was used to determine the average fluorescence intensity (A.U.) across a region of interest around the gray matter in maximum projection images. Values were normalized to the average value in control animals at 24 months and shown as the average ± SEM.

### Statistical analyses

**Fig. 2:**
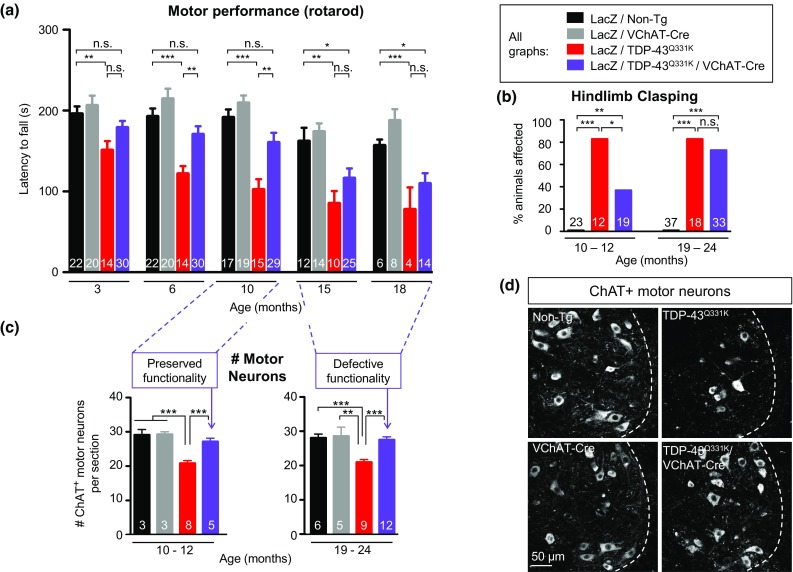
Reduction of mutant TDP-43 within motor neurons delays onset of motor impairment in TDP-43^Q331K^ mice, but not disease progression, despite age-dependent preservation of motor neuron numbers. **a** Motor performance (using rotarod) of TDP-43^Q331K^/VChAT-Cre animals was significantly improved compared to TDP-43^Q331K^ animals at 6 and 10 months of age, but declined by 15 months reaching poor motor performance close to TDP-43^Q331K^. Data are shown as average ± SEM with *n* = 10–30 mice per group for ages 3–15 months and *n* = 4–14 animals per genotype at 18 months. *P < 0.05, **P < 0.005, and ***P < 0.0001 using an unpaired *t* test. **b** The percentage of animals displaying a clasping phenotype was protected in TDP-43^Q331K^/VChAT-Cre animals at 10 months, but not at 19 months of age (at 10 months, *n* > 12 animals, and at 19 months, *n* > 18, in TDP-43^Q331K^ and TDP-43^Q331K^/VChAT-Cre animals, respectively). Statistical analysis was performed using Fisher’s exact test (*P = 0.025, **P < 0.005, ***P < 0.0001). **c**, **d** Motor neuron loss was rescued in TDP-43^Q331K^/VChAT-Cre animals compared to TDP-43^Q331K^ animals. **c** Quantification of ChAT positive motor neurons (average per section ± SEM, *n* ≥ 3 in controls, and *n* ≥ 5 animals in TDP-43^Q331K^ and TDP-43^Q331K^/VChAT-Cre groups; ***P ≤ 0.0006 and **P = 0.004). **d** Representative confocal micrograph of ChAT-positive α-motor neurons in lumbar spinal cords from TDP-43^Q331K^ transgenic mice at 19 months of age. *Scale bar* 50 µm. *n* ≥ 2 in controls, and *n* ≥ 3 animals in TDP-43^Q331K^ and TDP-43^Q331K^/VChAT-Cre groups

Differences between two groups were determined by an unpaired *t* test, using GraphPad Prism Software. The threshold for significance was set as *P* ≤ 0.05. Alternatively, Fisher’s exact test was used to determine significance for the percentage of animals affected by hindlimb clasping (Fig. 2b).

## Results

### Motor neuron-specific reduction of mutant TDP-43 in mice expressing mutant TDP-43 broadly throughout the central nervous system

Transgenic mice expressing a Myc-tagged ALS-linked Q331K mutant human TDP-43 at 1.2 times the normal level of TDP-43 throughout the central nervous system, including in motor neurons, oligodendrocytes and astrocytes, were previously shown to develop progressive adult-onset motor neuron disease [2]. The TDP-43^Q331K^ transgene (flanked by loxP sites) is inactivatable by action of the Cre recombinase. To test the degree to which motor phenotypes were caused by cell intrinsic damage from mutant TDP-43 expressed within motor neurons, the TDP-43^Q331K^ animals were bred to VChAT-Cre mice that have previously been shown to postnatally express Cre recombinase selectively within motor neurons [40, 69] (Fig. 1a). Excision efficiency and specificity of the TDP-43^Q331K^ transgene was initially determined using the Rosa26 reporter strain which ubiquitously expresses a β-galactosidase transgene preceded by a floxed stop cassette and only expresses β-galactosidase in cells in which Cre recombinase has previously been activated [59]. By 2 months of age, Cre-mediated recombination in TDP-43^Q331K^/VChAT-Cre/Rosa26 mice was seen in 50% of choline acetyltransferase (ChAT)-positive motor neurons throughout the lumbar spinal cord (Fig. 1b, c, Supplementary Fig. 1a, b), consistent with previous reports [40]. Also as expected, Cre-activated excision was selectively found only within motor neurons, with β-galactosidase activity restricted to ventral horn motor neurons (Supplementary Fig. 1a).

Finally, by evaluating the expression pattern of mutant TDP-43 in the ventral horn of lumbar spinal cords, excision efficiency of the actual TDP-43^Q331K^ transgene was measured to be 45% (Fig. 1d, e, Supplementary Fig. 1c, d) in TDP-43^Q331K^/VChAT-Cre animals. Excision was selective to motor neurons, as no excision of TDP-43^Q331K^ was found in sensory neurons in the dorsal horn or glial cells (Supplementary Fig. 1e). VChAT-Cre inactivation of the TDP-43^Q331K^ transgene did not significantly alter overall levels of mutant TDP-43 expression within the central nervous system (CNS), as determined by measuring TDP-43 mRNA (Fig. 1f) and protein (Fig. 1g) levels in total spinal cord extracts. Consistent with previous reports establishing a mechanism for TDP-43 autoregulation [3, 4, 48], the levels of endogenous mouse TDP-43 were reduced compared to non-transgenic animals.

### Motor neuron-specific reduction of mutant TDP-43 levels delays onset of motor deficits

To determine the contribution of mutant TDP-43-dependent damage within motor neurons to disease pathogenesis, motor performance was evaluated longitudinally. Age-dependent motor deficits in TDP-43^Q331K^ mice (relative to non-transgenic littermates) initiated prior to 3 months of age and the deficits increased during aging (Fig. 2a), as previously reported [2]. In contrast, the motor performance of TDP-43^Q331K^/VChAT-Cre animals was nearly unaffected up to 10 months of age (Fig. 2a, Supplementary Fig. 2a, with a latency to fall of 160 s, compared to 191 s in non-transgenic animals, *n* = 29 and 17, respectively). Reduction of TDP-43^Q331K^ synthesis within motor neurons delayed the appearance of a hindlimb clasping motor phenotype that developed in >80% of TDP-43^Q331K^ animals by 10 months (Fig. 2b). Thus, motor neuron-specific reduction of mutant TDP-43 delays onset of significant motor deficits.

### Reduction of mutant TDP-43 within motor neurons prevents age-dependent motor neuron death, but only modestly delays degeneration of motor axons and neuromuscular junctions

Although disease initiation was delayed, disease in the TDP-43^Q331K^/VChAT-Cre animals reached a similar disease state to the TDP-43^Q331K^ mice by 18 months of age (Fig. 2a, Supplementary Fig. 2a). Nevertheless, eliminating TDP-43^Q331K^ synthesis within individual motor neurons almost completely prevented the 30% loss of lumbar spinal cord α-motor neurons (27 ± 1 ChAT-positive motor neurons per section in TDP-43^Q331K^/VChAT-Cre, *n* = 12, compared with 21 ± 1 ChAT-positive motor neurons in TDP-43^Q331K^, *n* = 9, *P* < 0.001 and 28 ± 1 ChAT-positive motor neurons in non-transgenic littermates, *n* = 6) (Fig. 2c, d).

**Fig. 3:**
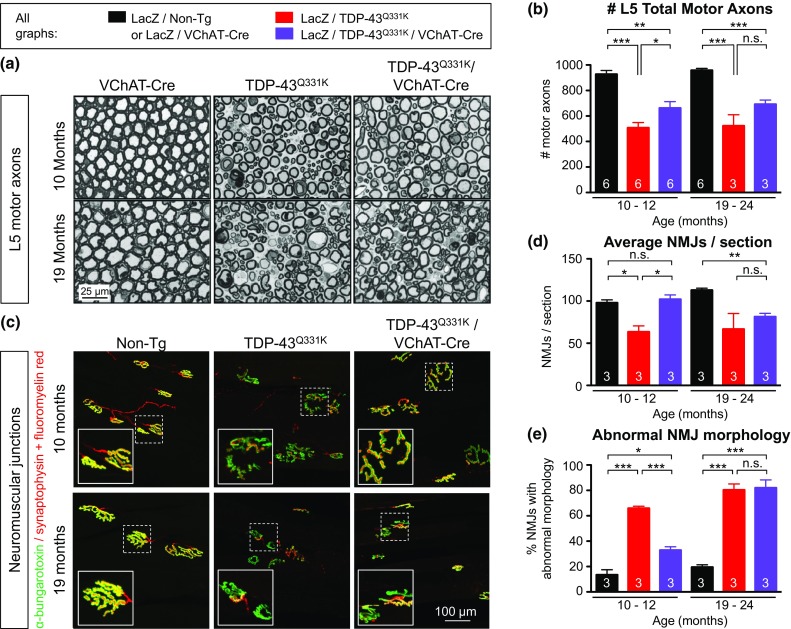
Reduction of mutant TDP-43 within motor neurons only partially delays degeneration of motor axons and neuromuscular junctions. **a** Representative micrograph of motor axons in the lumbar L5 motor root at 10 or 19 months of age. *Scale bar* 25 µm. **b** Quantification of total motor axons in the lumbar L5 motor root at 10 or 19 months of age (average ± SEM, *n* = 6 in controls, and *n* ≥ 3 animals in TDP-43^Q331K^ and TDP-43^Q331K^/VChAT-Cre groups; *P < 0.04, **P < 0.009, and ***P = 0.0001 using an unpaired *t* test). **c** Representative neuromuscular junctions from non-transgenic, TDP-43^Q331K^ and TDP-43^Q331K^/VChAT-Cre animals. Muscle acetylcholine receptor endplates were labeled with α-bungarotoxin, and motor axons (synaptic motor axon terminals and myelinated axons) were marked by synaptophysin antibody and fluoromyelin red, respectively. *Scale bar* 100 µm. **d** Quantification of average neuromuscular junctions per section ± SEM, *n* = 3; *P < 0.04, **P < 0.003. **e** Assessment of neuromuscular junction morphology during aging. Morphology was scored in >300 NMJs per animal, and abnormal morphology included fragmented or small NMJs; average ± SEM, *n* = 3; *P < 0.02, ***P < 0.0006

Despite complete rescue of motor neuron cell bodies, however, degeneration of motor axons (measured in L5 roots) was only modestly inhibited in TDP-43^Q331K^/VChAT-Cre animals compared to TDP-43^Q331K^ mice, both at onset and at later stages of disease (averages of 662 and 508 motor axons, respectively, compared to 921 in non-transgenic controls, *n* = 6, *P* < 0.001 for TDP-43^Q331K^ compared to controls, or *P* < 0.04 for TDP-43^Q331K^/VChAT-Cre compared to TDP-43^Q331K^ at 10 months) (Fig. 3a, b, Supplementary Fig. 2b, c). Examination of the remaining motor axons revealed degenerating axons, vacuolization, and myelin defects in both TDP-43^Q331K^ and TDP-43^Q331K^/VChAT-Cre animals (Fig. 3a), with a modest protection in TDP-43^Q331K^/VChAT-Cre animals at onset, but this benefit was not sustained with aging (Fig. 3a). Mutant TDP-43-dependent loss of the large-caliber α-motor axons (with axonal size >5.5 µm) was only partially alleviated at 10 months in TDP-43^Q331K^/VChAT-Cre and did not reach numbers close to those in non-transgenic animals (Supplementary Fig. 2b). Furthermore, the age-dependent atrophy of the remaining α-motor axons was similar to that in TDP-43^Q331K^ (Supplementary Fig. 2c).

Quantification of postsynaptic neuromuscular junctions in the gastrocnemius muscle using α-bungarotoxin staining revealed a significant degree of protection in TDP-43^Q331K^/VChAT-Cre animals at 10–12 months, in contrast to the 35% reduction in the number of neuromuscular endplates seen in TDP-43^Q331K^ animals (Fig. 3d, *n* = 3, *P* < 0.02 for TDP-43^Q331K^ compared to controls, and *P* < 0.02 for TDP-43^Q331K^/VChAT-Cre compared to TDP-43^Q331K^). However, this protection was not sustained at 19–24 months (Fig. 3c, d, *n* = 3, *P* < 0.003 for TDP-43^Q331K^/VChAT-Cre compared to controls). Morphological examination of neuromuscular junctions further indicated abnormalities including fragmented or small endplates in TDP-43^Q331K^ animals (Fig. 3e), which were significantly attenuated at 10–12 months but not prevented at later stages of disease in TDP-43^Q331K^/VChAT-Cre animals (Fig. 3e, 33% of NMJs with aberrant morphology in TDP-43^Q331K^/VChAT-Cre compared to 65% in TDP-43^Q331K^, *n* = 3, *P* < 0.0005 at 10–12 months of age). Therefore, motor neuron-specific reduction of mutant TDP-43^Q331K^ was not sufficient to abrogate defects in motor axons, neuromuscular junctions, and behavioral performance at late stages of disease, despite nearly complete preservation of motor neuron cell bodies.

### TDP-43^Q331K^ expression within motor neurons contributes to nuclear aberrations

**Fig. 4:**
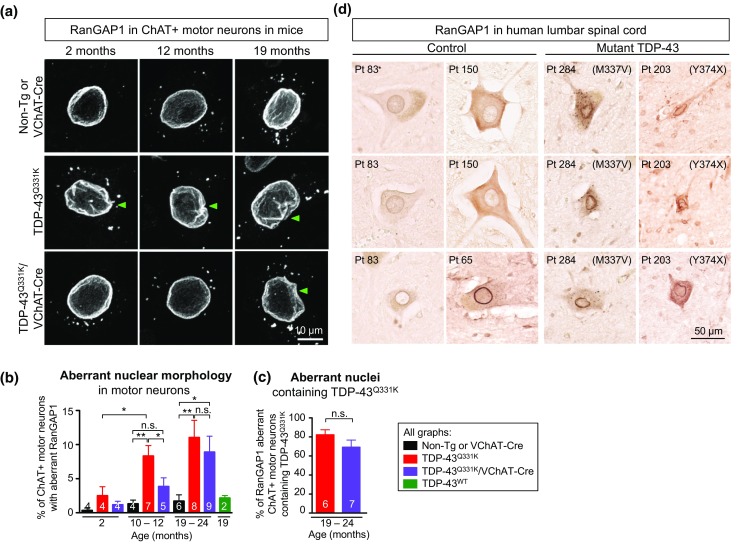
The TDP-43 mutant-dependent nuclear morphology defects in motor neurons of mice and ALS patients harboring TDP-43 mutations are delayed upon reduction of mutant TDP-43 within mouse motor neurons. **a**, **b** Age-dependent increased nuclear morphology aberrations in motor neurons from mutant TDP-43^Q331K^ mice, which was significantly delayed but not prevented with disease progression upon motor neuron specific excision of the transgene in TDP-43^Q331K^/VChAT-Cre animals. **a** Representative confocal micrographs of nuclear membrane morphology in lumbar spinal cord motor neurons, revealed by RanGAP1 immunostaining in ChAT-positive cells. *Green arrowheads* indicate examples of motor neurons with nuclear aberrations. *Scale bar* 10 µm. **b** Quantification of ChAT positive motor neurons with aberrant nuclear morphology revealed by RanGAP1 immunostaining (average ± SEM, *n* ≥ 4 animals per genotype at each timepoint). **c** Quantification of aberrant RanGAP1 containing nuclei that are TDP-43^Q331K^ positive in lumbar spinal cord motor neurons at 19–24 months (*n* ≥ 6 animals from each group in non-transgenic, TDP-43^Q331K^, and TDP-43^Q331K^/VChAT-Cre). **d** Aberrant nuclear ring shape in motor neurons in the lumbar spinal cord anterior horn of ALS patients harboring TDP-43 mutations. RanGAP1 immunostaining shows an irregular nuclear membrane in motor neurons from mutant TDP-43 patients (*n* = 2), compared to large round nuclei in motor neurons from control patients (*n* = 3). *Scale bar* 50 µm

Defects in nuclear membrane structure or nucleocytoplasmic transport have been reported in SOD1-mediated toxicity in the CNS of ALS patients [33] and mouse models [42, 70], and more recently in disease caused by the hexanucleotide repeat expansion in *C9orf72* [17, 27, 71, 72]. Given the diminished motor performance in aged TDP-43^Q331K^/VChAT-Cre animals despite absence of motor neuron death, we examined the integrity of the nuclear membrane in the remaining motor neurons. Age-dependent aberrations in nuclear morphology, including nuclear membrane invaginations (Fig. 4a, green arrows), were observed as early as 2 months of age in lumbar spinal cord motor neurons of TDP-43^Q331K^ animals compared to non-transgenic mice (*n* = 4) (Fig. 4a, b, Supplementary Fig. 3a and movie 1).

In contrast, motor neuron-specific reduction of mutant TDP-43^Q331K^ significantly delayed the appearance of RanGAP1 aberrations in ChAT positive motor neurons of TDP-43^Q331K^/VChAT-Cre animals (Fig. 4a, b; 8.5% in TDP-43^Q331K^ vs 3.8%, in TDP-43^Q331K^/VChAT-Cre, *n* = 7 vs 5 animals, respectively, *P* = 0.043). Nevertheless, by 19 months, the extent of motor neurons with aberrant nuclear morphology (scored as shown in Fig. 4a, green arrows), in the TDP-43^Q331K^/VChAT-Cre mice was almost equivalent to that of TDP-43^Q331K^ animals (Fig. 4b; 11 and 9%, respectively; *n* ≥ 8), thus correlating with severity of the motor performance (Fig. 2a). Most of the motor neurons with RanGAP1 aberrations (including invaginations in the nuclear membrane–Fig. 4a, Supplementary Fig. 3a; movie 1) expressed TDP-43^Q331K^ (Fig. 4c, Supplementary Fig. 3b, green arrow). Nevertheless, one-fourth of the motor neurons with aberrations in RanGAP1 lacked mutant TDP-43 (Supplementary Fig. 3b, white arrows). In animals expressing wild-type human TDP-43 at total levels close to endogenous in a normal mouse [2], nearly all motor neurons maintained normal nuclei at 19 months of age (Fig. 4b, green bar), as in non-transgenic animals (Supplementary Fig. 3b). Cytosolic foci containing RanGAP1 were present in young animals, and appeared to increase in size during aging (Fig. 4a), but the number of motor neurons containing foci did not differ between genotypes or during disease progression (Fig. 4a, Supplementary Fig. 3c).

Defects in nuclear membrane morphology were also found in lumbar spinal cords of ALS patients harboring a mutation in TDP-43 (Fig. 4d). Immunohistochemistry for RanGAP1 in the anterior horn revealed a smaller irregular ring shape around nuclei in mutant TDP-43 patients (*n* = 2), compared to large round nuclear membrane in motor neurons from controls (*n* = 3). Consistent with observations in animals (Fig. 4a), cytosolic RanGAP1-positive foci were observed in both patients and controls, and an increase in their size appears to correlate with increased age (Fig. 4d). Together, these data suggest that nuclear membrane morphology, as visualized by RanGAP1 staining, can distinguish healthy motor neurons from degenerating motor neurons, and these defects correlate with poor functionality of surviving motor neurons in TDP-43^Q331K^/VChAT-Cre animals at late stages of disease.

### Reduction of mutant TDP-43^Q331K^ within motor neurons does not alter neuroinflammation

**Fig. 5:**
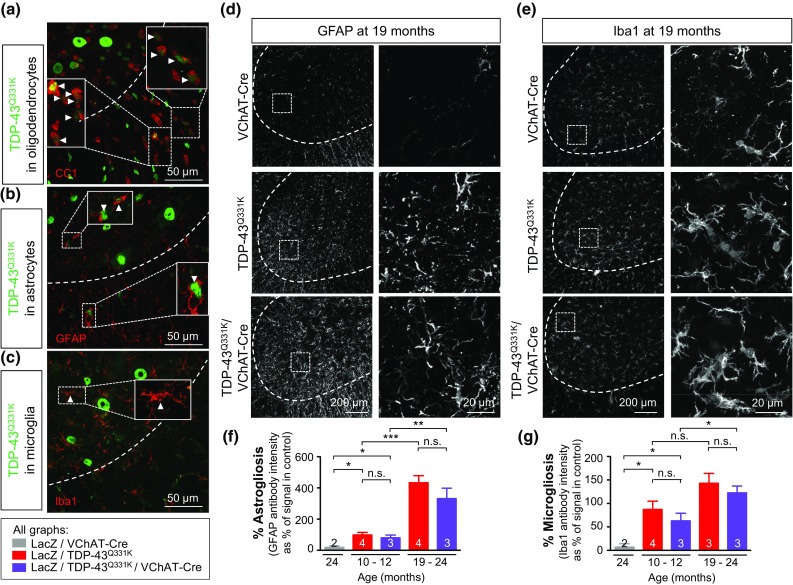
A non-cell autonomous contribution of mutant TDP-43^Q331K^ to disease progression: reduction of mutant TDP-43^Q331K^ within motor neurons does not alter neuroinflammation during disease progression. **a** Broad expression of the human TDP-43^Q331K^ transgene in glial cells including oligodendrocytes and astrocytes, but not microglia. Oligodendrocytes (CC1 antibody in (**a**), astrocytes (GFAP antibody in (**b**), and microglia (Iba1 antibody in (**c**) were identified by immunofluorescence in spinal cords from 12-month-old TDP-43^Q331K^ animals using antibodies for cell type-specific markers. *Arrows* highlight glial cells (in *red*) containing TDP-43^Q331K^ positive nuclei (in *green*). *Dashed outlines* correspond to the boundary between *gray* and *white* matter. *Scale bar* 50 μm. **d**, **e** Representative micrographs of lumbar spinal cords from VChAT-Cre, TDP-43^Q331K^ and TDP-43^Q331K^/VChAT-Cre animals at late stages of disease processed for immunofluorescence using an antibody detecting activated astrocytes (GFAP) (**d**) or activated microglia (Iba1) (**e**). *Scale bar* 200 μm, with enlarged inset panels on the *right*. **f**, **g** Astrogliosis and microgliosis were quantified by measuring GFAP (**f**) or Iba1 (**g**) fluorescence intensity from confocal images in the lumbar spinal cord of 12 or 19-month-old animals (*n* ≥ 3 in TDP-43^Q331K^ and TDP-43^Q331K^/VChAT-Cre; *P < 0.05, **P < 0.005, and ***P < 0.0001 using an unpaired *t* test)

TDP-43^Q331K^ was widely expressed in the CNS and accumulated in most oligodendrocytes (Fig. 5a, arrows) and astrocytes (Fig. 5b, arrows), but not microglia (Fig. 5c) (scored by immunofluorescence using the cell-type specific markers CC1, GFAP and Iba1, respectively). Remarkably, age-dependent activation of astrocytes (Fig. 5d) and microglia (Fig. 5e) in ventral horn of spinal cords of TDP-43^Q331K^ animals (scored by GFAP and Iba1 immunoreactivity, respectively; Fig. 5f, g) was indistinguishable irrespective of whether mutant TDP-43 was expressed by motor neurons or not, both at young and old ages.

## Discussion

One of the key discoveries of the past decade is that ALS-like disease pathogenesis from ubiquitously expressed mutant SOD1 is non-cell autonomous [9]. Mutant SOD1 synthesis within motor neurons [9, 69] and precursors of oligodendrocytes [30] drives disease onset, but mutant-mediated damage within astrocytes [67, 69] and microglia [5, 9] drives rapid disease progression. To this, we have now established that ALS-mutant linked TDP-43 mutations expressed at moderate levels in a pattern mimicking endogenous TDP-43 also cause toxicity in a non-cell autonomous manner. Eliminating mutant TDP-43^Q331K^ synthesis in a proportion of motor neurons delayed disease onset, reduced aberrant nuclear morphology in those neurons at early disease stages, and almost eliminated age-dependent accelerated death of those motor neurons. Nevertheless, despite nearly complete rescue of motor neuron loss, deficits in motor performance at later disease stages—accompanied by motor axon degeneration, neuromuscular junction loss, and increased nuclear morphology aberrations within motor neurons—were unaffected, as was the timing of sustained activation of both astrocytes and microglia.

Our findings add to the growing body of evidence uncoupling motor neuron cell death from overall ALS disease course. In mutant SOD1 mouse models, genetic interventions which successfully prevented motor neuron cell death either by modulating levels of apoptotic factors [19, 54], elevating chaperones [52], or by rescuing mitochondrial calcium buffering capacity [47], were ineffective in altering disease course or prolonging lifespan. Similarly, rescuing motor neuron death in mutant FUS-mediated disease does prevent motor deficits and axonal damage during aging [55].

A convergence of recent evidence has implicated errors in nucleo/cytoplasmic transport as a component of normal aging [11] as well as several neurodegenerative disorders [13], including ALS/FTD [8, 49], Huntington’s disease (Cleveland and Lagier-Tourenne, personal communication) [35, 43, 68], and other repeat expansion models [38, 51]. Studies in yeast [27] and flies [61] have identified components of nuclear transport as modifiers of toxicity in models expressing mutant TDP-43, or the *C9orf72* hexanucleotide repeat expansion [17, 71]. Altered distribution of importins has been described in motor neurons of mice expressing ALS-linked mutation in SOD1 [70], as have aberrations in RanGAP1 localization in *C9orf72* patients [71] and irregularities in the nuclear membrane of cells with TDP-43 pathology in sporadic ALS patients [33]. Mutations in the mRNA nuclear export factor Gle1 have also been identified in rare cases of ALS [28], and recently expression of TDP-43 fragments prone to aggregation has been reported to cause mRNA retention in the nuclei of cultured cells [68].

To these earlier efforts, our findings suggest that TDP-43 aggregation is not required to drive nuclear morphology aberrations as accumulation of mutant TDP-43 to only about the normal level of endogenously expressed TDP-43 in both neurons and glial cells drives progressive motor neuron disease with specific loss of a third of lower motor neurons, despite the absence of cytosolic accumulation of protein aggregates (nuclear or cytoplasmic) and loss of nuclear TDP-43 at any age [2]. Thus, our data provide in vivo evidence that 1) ALS-linked mutant TDP-43 causes age-dependent defects in nuclear membrane morphology (as indicated by RanGAP1 staining) not only in mouse motor neurons (correlating with motor functionality), but also in ALS patients harboring TDP-43 mutations, and 2) motor neuron-specific reduction of mutant TDP-43 significantly delays the appearance of such defects and of motor performance deficits. It is noteworthy that nuclear aberrations have also been reported in other ALS model systems [17, 27, 33, 42, 70–72], as well as other neurodegenerative diseases including Huntington’s disease (Cleveland and Lagier-Tourenne, personal communication), suggesting that nuclear morphology aberrations could be a general hallmark of neurodegeneration, possibly indicative of dysfunctional neurons before their degeneration. How ALS-linked mutations in TDP-43 provoke alterations in RanGAP1 distribution is not established, and additional efforts are now needed to determine the underlying mechanism(s) and to test whether those are widely applicable to sporadic disease.

Prior studies have established non-cell autonomous toxicity of mutant SOD1 from microglial cells [6, 9], oligodendrocytes [29, 30], and astrocytes [67, 69] (reviewed in Ref. [25]). Whether non-cell autonomous neuronal-glial signaling is critical in TDP-43 causing disease is not yet established. While mutant astrocytes (derived from glial precursors isolated from transgenic TDP-43^A315T^ mice [20] or from patient-induced pluripotent stem cells (iPSCs) harboring the TDP-43^M337V^ mutation [57]) were not toxic to co-cultured wild-type mouse motor neurons, conditioned medium from TDP-43^A315T^ mouse astrocytes [53] or microglia treated with recombinant TDP-43 mutants [73] triggered motor neuron death. Additionally, mutant TDP-43-mediated toxicity in mutant TDP-43 motor neurons was not enhanced by co-cultured mutant astrocytes derived from iPSCs [57]. However, while transplanted glial precursor cells expressing mutant TDP-43^A315T^ had no effect on motor neuron death or behavioral phenotypes in wild-type rats [20], astrocyte-specific expression of mutant TDP-43^M337V^ caused rapid fatal motor deficits within a month after induction of the transgene in transgenic rats [64]. Similarly, widespread overexpression of wild-type TDP-43 through an AAV9-mediated viral intravenous delivery in neonatal rats provoked rapid mortality in less than 1 month with only a mild (14%) loss of motor neurons, consistent with TDP-43-dependent alterations in motor neuron functionality independent of cell death, and/or contribution(s) of the surrounding cells including glia [65].

Our evidence strongly supports that mutant TDP-43-mediated disease is non-cell autonomous. While most of the motor neurons with nuclear defects were mutant TDP-43 positive, one-fourth of them did not express the mutant protein, suggesting that damage to those nuclei is mediated by mutant TDP-43 expression in other cell types surrounding the motor neurons. Given the wide expression of mutant TDP-43, and that astrogliosis and microgliosis were not attenuated by mutant TDP-43 gene excision from motor neurons, toxicity must also be mediated from a mutant acting in non-motor neurons as a contributor to motor deficits associated with age. Similarly, FUS-mediated disease in mice where one copy of endogenous FUS was replaced by a mutant form of FUS restricted to the cytoplasm is also non-cell autonomous [55]. Altogether, non-cell autonomous toxicity is likely to be widely applicable in ALS pathogenesis. Perhaps most importantly, therapies specifically targeting motor neurons have promise to delay early stages of disease, but it seems unlikely that they will suffice to extend overall survival, unless non-neuronal cells are also targeted.

The mechanisms underlying non-cell autonomous toxicity mediated by mutant TDP-43 are not known. TDP-43 has been reported to be secreted via exosomes from neurons [24], which could corroborate the hypothesis, so far untested in vivo, that TDP-43 proteinopathy propagates cell to cell [45]. TDP-43 proteins can be detected in CSF of ALS patients [18, 31, 46], suggesting that TDP-43 may be present in the CNS extracellular space. Addition of mutant TDP-43 protein fragments to cultured microglia did induce a receptor-mediated proinflammatory signaling response which was neurotoxic [73] possibly through passive diffusion of TDP-43 [upon cleavage and/or post-translation modification] or release extracellularly during cell death [58]. However, the latter pathway is unlikely as motor neuron death was prevented in our TDP-43^Q331K^ animals even at late ages. One underlying mechanism would be that mutant TDP-43 expressing glial cells and/or other surrounding neurons may release yet unidentified factors that are selectively toxic for motor neurons, as has been proposed in mutant SOD1-mediated disease [39, 41]. Another alternative mechanism for the non-cell autonomous component to disease progression could be altered neuronal connectivity, given the expression of mutant TDP-43 within other neuronal sub-types and widespread splicing defects [2].

## Electronic supplementary material

Below is the link to the electronic supplementary material.

Supplementary material 1 (AVI 481035 kb)

Supplementary material 2 (PDF 15138 kb)
