# Supplementary material for: Mutant TDP-43 within motor neurons drives disease onset but not progression in amyotrophic lateral sclerosis
Source: Acta Neuropathol. 2017 Mar 29;133(6):907–22. doi: 10.1007/s00401-017-1698-6 (PMC5427168; doi:10.1007/s00401-017-1698-6)
Supplement: Supplementary file 2 — Supplementary material 2 (PDF 15138 kb) [file 401_2017_1698_MOESM2_ESM.pdf]

Mutant TDP-43 within motor neurons drives disease onset but not progression in Amyotrophic Lateral Sclerosis

Dara Ditsworth[1], Marcus Maldonado[1], Melissa McAlonis-Downes[1], Shuying Sun[1,5], Amanda Seelman [1], Kevin Drenner[1], Eveline Arnold[1], Shuo-Chien Ling[1,6,7], Donald Pizzo[2], John Ravits[3], Don W. Cleveland[1,3,4,*], and Sandrine Da Cruz[1,*]

ELECTRONIC SUPPLEMENTARY MATERIALS

Contents	Page Number
Supplementary Methods	1 – 2
Supplementary Figure Legends	3 – 4
Supplementary Figures 1 – 3	5 – 7
Supplementary Movie 1	Online

SUPPLEMENTARY Methods:

Primers for genotyping or qRT-PCR:

Genotyping primers are below:

Cre Primer set #1: CTG CAT TAC CGG TG ATG CA

Cre Primer set #2: ACG TTC ACC GGC ATC AAC GT

Expected 500bp product in Cre positive animals, no band in negatives

huTDP-43 primer set #1 (exon 2F): AGAGGTGTCCGGCTGGTAG

huTDP-43 primer set #2 (exon 3R): CCTGCACCATAAGAACTTCTCC

moTDP-43 primer set #3 (3UTR 1F): TTTTCATACACGGCGGTACA

moTDP-43 primer set #4 (3UTR 1R): GCCGCTCATGCTGTATATGA

Expected size for hTDP with DD1/2: 228bp in human TDP-43^{Q331K} animals

Expected size for mTDP with DD3/4: 371bp in all animals

LacZ Primer set #1: CCTGCGATGTCGGTTTCC G

LacZ Primer set #2: CGTATTCGCAAAGGATCAGC

The following primer sequences were used for qRT-PCR shown in Fig. 1f:

m/h TDP-4F	CAAGTGAAAGTAATGTCACA
m/h TDP-5R	TTTCTGCTTCTCAAAGGCTC
mTDP-4F	ATCTTAAAACTGGTCACTCG
mTDP-5R	AGACATCTACCACTTCTCCA
m Actg1g F	TGGATCAGCAAGCAGGAGTATG
m Actg1g R	CCTGCTCAGTCCATCTAGAAGCA
mRPS9 F	GACCAGGAGCTAAAGTTGATTGGA
mRPS9 R	GCGTCAACAGCTCCCGGGC

Primary antibodies:

The following primary antibodies were used for immunostaining:

β-gal (Promega Z3783, Mo, 1:500)

CC1 (Calbiochem, Mo, 1:500)

ChAT (Millipore Ab144P, Gt, 1:300)

GFAP (Dako Z0334, Rb, 1:1000)

GFAP (Millipore MAB360 clone GA5, Mo, 1:500)

Iba1 (Wako 019-19741, Rb, 1:500)

Myc (Millipore 4A6, 05-724, 1:600-1000)

Myc (Sigma C3956, Rb, 1:1000)

NeuN-488 (Millipore MAB377, Mo, 1:1000)

Nucleolin (Abcam Ab22758, Rb 1:1000)

RanGAP1 (Santa Cruz H-180, sc25630, Rb, 1:500)

RanGAP1 (Santa Cruz N-19, sc1862, Gt, 1:300)

RanGAP1 (Abcam ab92360, Rb, 1:300)

The following primary antibodies were used for immunoblotting:

hu/moTDP-43 (Proteintech #12892 Rb, 1:1000)

Hsp90 (Cell Signaling C45B5 #4877, Rb, 1:1000)

Myc-tag (Millipore 4A6, 05-724, Mo, 1:1000)

SUPPLEMENTARY FIGURE LEGENDS

Supplementary Fig. 1: Selective TDP-43^{Q331K} excision in motor neurons: VChAT-Cre activity in the lumbar spinal cord.

(**a**) β-galactosidase (β-gal) activity specifically detected in lumbar motor neurons of 2-month old LacZ/VChAT-Cre and LacZ/TDP-43^{Q331K}/VChAT-Cre animals following incubation with X-gal (blue). Scale bar, 200 μm. (**b**) Quantification of the β-gal positive cells ($n \geq 3$ per group). (**c-d**) Cre-excision of TDP-43^{Q331K} in about 45% of motor neurons was sustained during aging. **(c)** Representative micrographs from 12-month old animals showing TDP-43^{Q331K} in motor neurons by immunostaining with antibodies against the Myc tag (red) and ChAT (green). Scale bar, 200 μm. (**d**) Quantification of TDP-43^{Q331K} immunostaining in ChAT positive motor neurons in lumbar spinal cord at 12 months of age ($n \geq 3$ animals per genotype at 10-12 months). (**e**) Representative micrographs of dorsal spinal cord sections from 12-month old TDP-43^{Q331K} and TDP-43^{Q331K}/VChAT-Cre animals processed for immunofluorescence using antibodies for Myc (transgene positive cells), nucleolin (as nuclear marker) and NeuN (neuronal marker) revealing similar levels of TDP-43^{Q331K} expression between the two genotypes. Scale bar, 200 μm.

Supplementary Fig. 2: Reduction of mutant TDP-43 within motor neurons partially alleviates age-dependent motor axon degeneration in TDP-43^{Q331K} mice.

(**a**) Motor performance of TDP-43^{Q331K}/VChAT-Cre animals was nearly unaffected up to 10 months of age, but accelerated after disease onset reaching a similar disease state to the TDP-43^{Q331K} mice by 18 months of age. Motor deficits were measured by rotarod performance over time for TDP-43^{Q331K} and TDP-43^{Q331K}/VChAT-Cre groups (data are shown as average ± SEM, ** $P < 0.005$ using an unpaired t-test, *n* as shown in bar graph in **Figure 2a**). (**b-c**) Age-dependent motor axon degeneration was only partially alleviated in TDP-43^{Q331K}/VChAT-Cre compared to age-matched control mice. The distribution of L5 motor axon diameters shows a loss of large caliber motor axons in TDP-43^{Q331K} animals at 10-12 months (**b**) and further axonal atrophy at 19-22 months of age (**c**), corresponding to loss of α-motor axons with only partial preservation of number of motor axons at each timepoint in TDP-43^{Q331K}/VChAT-Cre animals ($n = 2$ in control animals or $n = 6$ in TDP-43^{Q331K} and TDP-43^{Q331K}/VChAT-Cre groups at 10-12 months, and $n = 3$ per group at 19-22 months.

Supplementary Fig. 3: Aberrant nuclear morphology in motor neurons in TDP-43^{Q331K} mice.

(**a**) Maximum projection of confocal micrograph of a lumbar spinal cord motor neuron from a 12-month old TDP-43^{Q331K} animal (**movie 1**), revealing nuclear membrane invaginations in a motor neuron stained with RanGAP1 antibody. Scale bar, 10 µm. (**b**) Representative confocal micrographs of the lumbar spinal cord from 19-24-month old VChAT-Cre, TDP-43^{Q331K}, TDP-43^{Q331K}/VChAT-Cre, or TDP-43^{WT} animals processed for immunofluorescence using antibodies against RanGAP1 (to assess nuclear morphology aberrations) and Myc (assessing the human TDP-43 transgene). *Asterisks* indicate ChAT positive motor neurons with normal nuclear shape, either containing (*green*) or lacking (*white*) human TDP-43. *Green arrows* highlight aberrant nuclei containing TDP-43^{Q331K}, and *white arrows* point to TDP-43^{Q331K}-negative motor neurons with aberrant nuclear shape. Scale bar, 20 µm. (**c**) Quantification of ChAT positive motor neurons containing RanGAP1-positive cytosolic foci (examples shown in main Fig. 4a). No correlation was found between the presence of cytosolic RanGAP1 foci and the extent of nuclear morphology aberrations. Values shown are the average ± SEM, $n > 4$.

Movie 1: Aberrant nuclear morphology in motor neurons of TDP-43^{Q331K} animals

3D confocal reconstruction of a lumbar spinal cord motor neuron from a 12-month old TDP-43^{Q331K} animal, showing RanGAP1 staining aberrations in a ChAT-positive motor neuron as an animation through individual planes of a 15-µm thick Z-series.

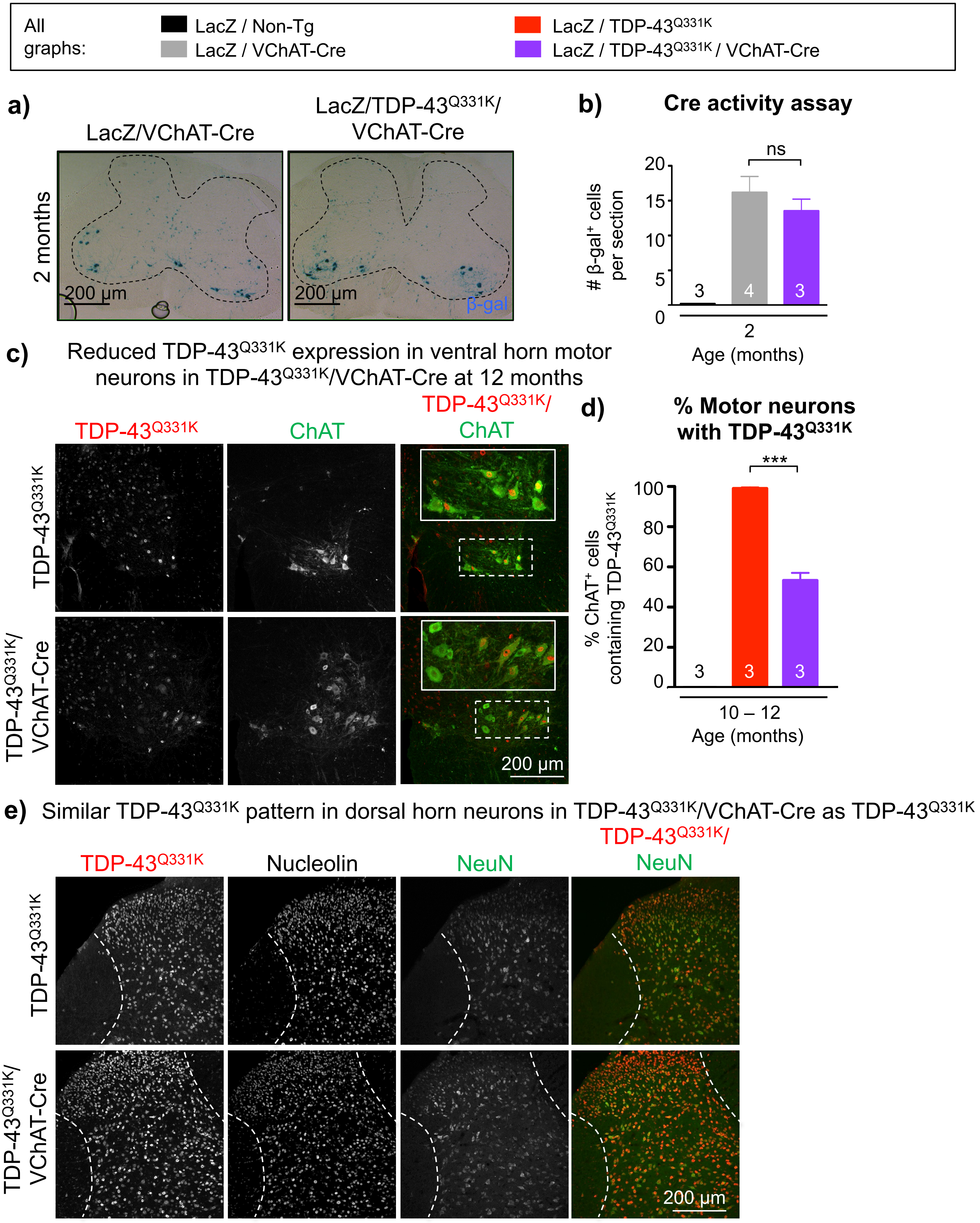

Supplementary Fig. 1: Selective TDP-43^{Q331K} excision in motor neurons: VChAT-Cre activity in the lumbar spinal cord

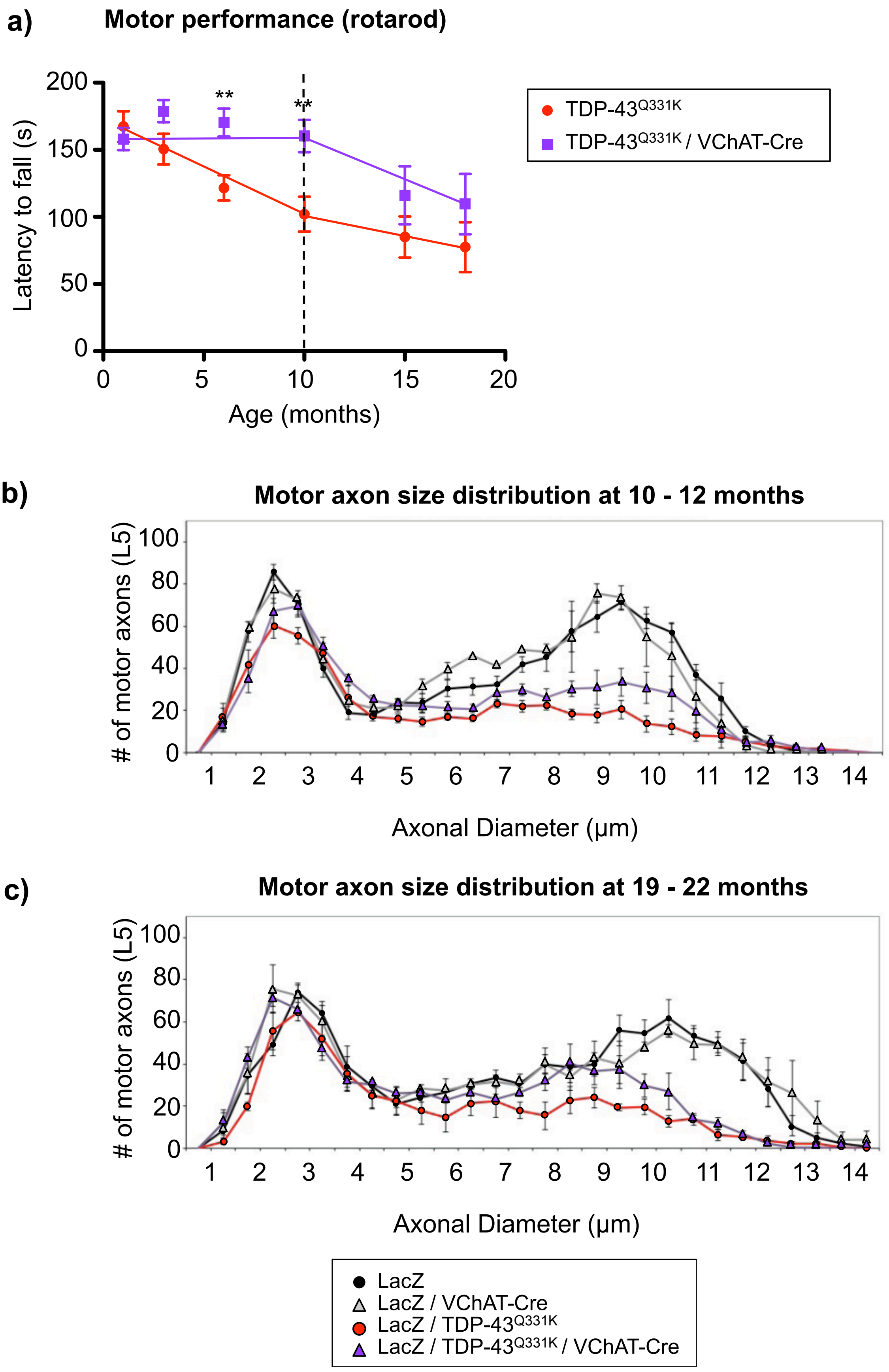

Supplementary Fig. 2: Reduction of mutant TDP-43 within motor neurons partially alleviates age-dependent motor axon degeneration in TDP-43^{Q331K} mice

Ditsworth et al

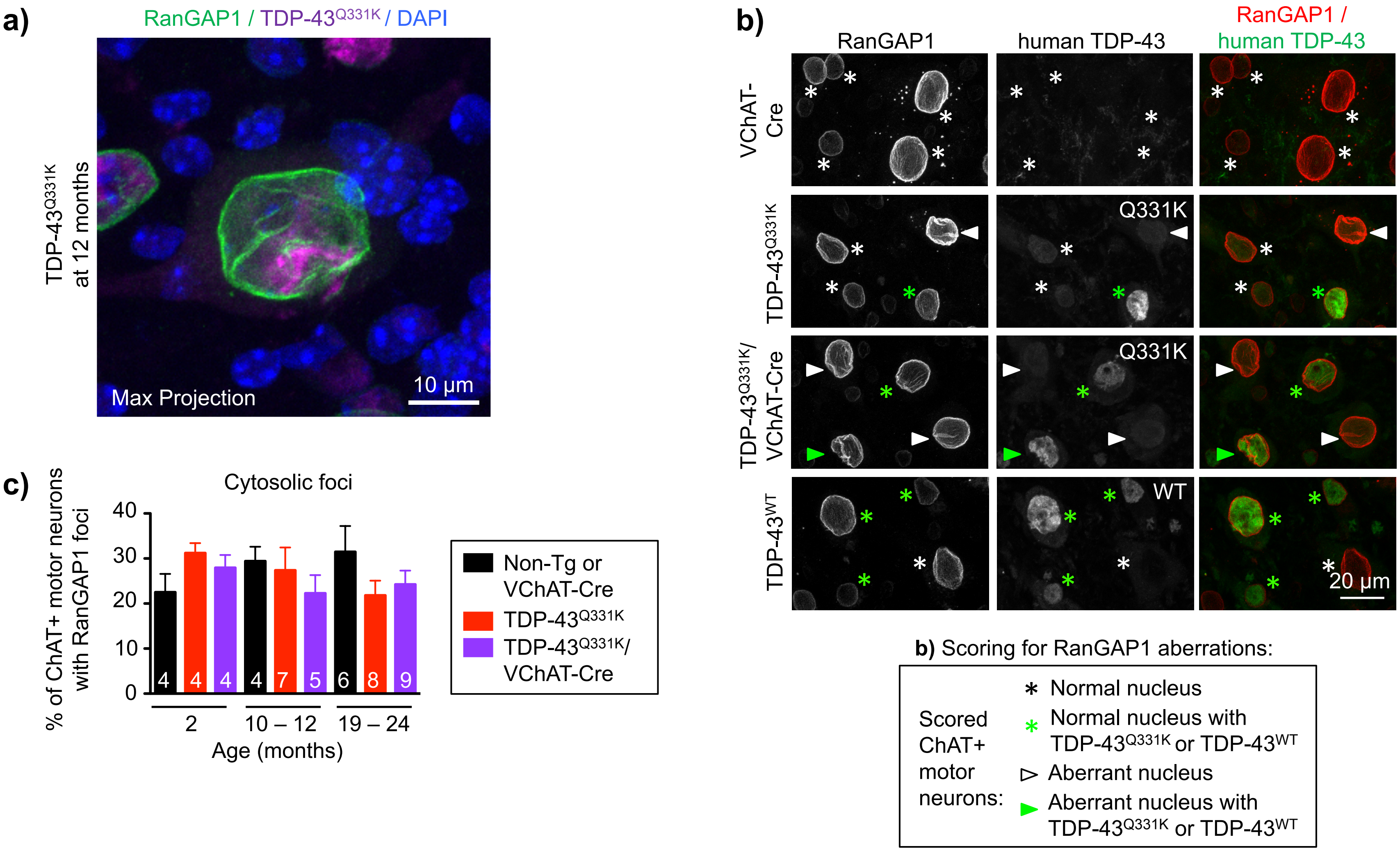

Supplementary Fig. 3: Aberrant nuclear morphology in motor neurons in TDP-43^{Q331K} mice
